# Polo-like kinase 1 as a biomarker predicts the prognosis and immunotherapy of breast invasive carcinoma patients

**DOI:** 10.32604/or.2023.030887

**Published:** 2023-12-28

**Authors:** JUAN SHEN, WEIYU ZHANG, QINQIN JIN, FUYU GONG, HEPING ZHANG, HONGLIANG XU, JIEJIE LI, HUI YAO, XIYA JIANG, YINTING YANG, LIN HONG, JIE MEI, YANG SONG, SHUGUANG ZHOU

**Affiliations:** 1 School of Big Data and Artificial Intelligence, Anhui Xinhua University, Hefei, 230088, China; 2 Department of Gynecology and Obstetrics, Maternity and Child Healthcare Hospital Affiliated to Anhui Medical University, Anhui Province Maternity and Child Healthcare Hospital, Hefei, 230001, China; 3 Department of Gynecology and Obstetrics, The Fifth Clinical College of Anhui Medical University, Hefei, 230032, China; 4 Departments of Breast Surgery, Fuyang Women and Children’s Hospital, Fuyang, 236000, China; 5 Departments of Pathology, Anhui Province Maternity and Child Health Hospital, Hefei, 230001, China; 6 Department of Pain, The First Affiliated Hospital of Anhui Medical University, Hefei, 230032, China; 7 Department of Gynecology and Obstetrics, Linquan Maternity and Child Healthcare Hospital, Fuyang, 236400, China

**Keywords:** Breast invasive carcinoma (BRCA), Polo-like kinase 1 (PLK 1), Random forest (RF), Support vector machine (SVM), Immune infiltration

## Abstract

**Background:**

Invasive breast carcinoma (BRCA) is associated with poor prognosis and high risk of mortality. Therefore, it is critical to identify novel biomarkers for the prognostic assessment of BRCA.

**Methods:**

The expression data of polo-like kinase 1 (PLK1) in BRCA and the corresponding clinical information were extracted from TCGA and GEO databases. PLK1 expression was validated in diverse breast cancer cell lines by quantitative real-time polymerase chain reaction (qRT-PCR) and western blotting. Single sample gene set enrichment analysis (ssGSEA) was performed to evaluate immune infiltration in the BRCA microenvironment, and the random forest (RF) and support vector machine (SVM) algorithms were used to screen for the hub infiltrating cells and calculate the immunophenoscore (IPS). The RF algorithm and COX regression model were applied to calculate survival risk scores based on the PLK1 expression and immune cell infiltration. Finally, a prognostic nomogram was constructed with the risk score and pathological stage, and its clinical potential was evaluated by plotting calibration charts and DCA curves. The application of the nomogram was further validated in an immunotherapy cohort.

**Results:**

PLK1 expression was significantly higher in the tumor samples in TCGA-BRCA cohort. Furthermore, PLK1 expression level, age and stage were identified as independent prognostic factors of BRCA. While the IPS was unaffected by PLK1 expression, the TMB and MATH scores were higher in the PLK1-high group, and the TIDE scores were higher for the PLK1-low patients. We also identified 6 immune cell types with high infiltration, along with 11 immune cell types with low infiltration in the PLK1-high tumors. A risk score was devised using PLK1 expression and hub immune cells, which predicted the prognosis of BRCA patients. In addition, a nomogram was constructed based on the risk score and pathological staging, and showed good predictive performance.

**Conclusions:**

PLK1 expression and immune cell infiltration can predict post-immunotherapy prognosis of BRCA patients.

## Introduction

Cancer is a major cause of mortality worldwide. Breast cancer is one of the most common malignancies among women that can lead to death. In fact, recent WHO data shows that breast cancer-related morbidity is about one-tenth of all newly diagnosed tumors, which has surpassed that of lung cancer [1]. Invasive breast carcinoma (BRCA) is a highly heterogeneous cancer that originates in the ductal epithelial cells of the breast and frequently metastasizes to distant organs, which is the major cause of mortality [2]. The average survival duration of patients with metastatic BRCA is only 18 to 30 months [3], and the most common sites of metastasis are the bone, lungs, and liver. Early screening through imaging and systemic adjuvant chemotherapy can reduce the risk of metastasis-related death. Currently, more than 80% of the BRCA patients undergo adjuvant chemotherapy, of which over 40% relapse and eventually die on account of metastasis [4,5]. Therefore, it is essential to explore effective and precise prognostic biomarkers in order to improve outcomes of BRCA patients and develop suitable treatment strategies.

Polo-like kinases (PLKs) are conserved filament/threonine protein kinases that play functional roles in DNA replication and cytoskeletal rearrangement during mitosis [6]. PLK1 is aberrantly expressed in various cancers and related to patient prognosis [7]. Recent studies have shown that PLK1 regulates the growth of breast cancer cells, and PLK1 inhibitors can be effective against BRCA. Wang et al. found that PLK1 blockade sensitized breast cancer cells to radiation by inhibiting autophagy [8]. In addition, the therapeutic effects of PLK1 inhibitors in BRCA patients have also been demonstrated [9]. Saatci et al. found that the combination of a PLK1 inhibitor with trastuzumab increased chemosensitivity of human epidermal growth factor receptor-2 (Her 2)-positive BRCA cells [10].

Machine learning algorithms, such as Random Forest (RF) and Support Vector Machine (SVM), are routinely used to analyze microarray data or large population gene sequencing data [11]. According to the statistical learning theories and structural risk minimization principles, SVM enables pattern identification and regression [12]. In the last few decades, machine learning algorithms have been used to predict prognostic biomarkers of various cancers [13], and can be applied to breast cancer as well.

To this end, we analyzed the expression level, clinical relevance and prognostic role of PLK1 in BRCA, and identified PLK1 expression as an independent risk factor. The immune landscape of BRCA was also assessed, and the COX regression method, and RF and SVM algorithms were used to determine the correlation between immune cell infiltration and PLK1 expression. Based on the aberrant immune cell populations and PLK1 expression, a prognostic signature for BRCA was established.

## Materials and Methods

### Data extraction

The transcriptomic and clinical data of BRCA patients were obtained from The Cancer Genome Atlas (TCGA) database. Immunotherapy data of the GSE173839 BRCA cohort was extracted from the Gene Expression Omnibus (GEO). The Molecular Taxonomy of Breast Cancer International Consortium (METABRIC) dataset was downloaded from the cBio cancer genomics portal (cBioPortal). Only samples with complete clinical and survival data were included in the analysis. The transcriptomic and clinical data of the IMvigor210 cohort were extracted using the “IMvigor210CoreBiologies” R package (http://research-pub.gene.com/IMvigor210CoreBiologies/packageVersions/). Ethics committee approval was not required since all data were obtained from public databases. The flow chart is shown in Suppl. Fig. 1.

### Machine learning methods

The decision trees were acquired by the RF algorithm with the “randomForest R” package, which obtained a set of averaged features by the complex interactions. In addition, the RF algorithm with the “randomForestSRC” R package was used to calculate the corresponding risk score (https://www.randomforestsrc.org/articles/survival.html). SVM was used for data classification and regression.

### Scoring of immunotherapy response

The pathological complete response (pCR) was evaluated as a surrogate of long-term prognosis. The immunophenoscore (IPS) was calculated using machine learning to quantify the immune response [14]; higher IPS score is indicative of greater tumor immunogenicity. The “mafools” R package was used to estimate tumor mutation burden (TMB). Tumor heterogeneity was assessed with the Mutant-Allele Tumor Heterogeneity (MATH) algorithm; greater MATH values indicate higher tumor heterogeneity. The immune checkpoint inhibitors (ICIs) were predicted using the Tumor Immune Dysfunction and Rejection (TIDE) algorithm [15].

### Immune cell infiltration analysis

Single sample gene set enrichment analysis (ssGSEA) was performed to evaluate the infiltration of different immune cell populations using the “GSVA” R package [16].

### Quantitative real-time polymerase chain reaction (qRT-PCR)

The normal breast epithelial cell line MCF10A, non-invasive BRCA cell lines BT-20, SK-BR-3 and MDA-MB-231, and the invasive BRCA cell line MCF-7 were purchased from American Type Culture Collection (ATCC, Manassas, VA, USA). The cells were cultured at 37°C with 5% CO_2_. Total RNA was extracted from the cultured cells, quantified and reverse transcribed to cDNA. The RT-PCR mix was prepared using the cDNA template (1 µL), forward and reverse primers (0.3 µL each), SYBR reagent (5 µL), and diethylpyrocarbonate (DEPC) water (3.4 µL). The reaction was performed on the BIO-RAD CFX cycler with the following conditions: 95°C for 5 min, and 39 cycles of 95°C for 10 s, 58°C for 30 s, and 72°C for 20 s. The relative gene expression level was calculated using the 2^−ΔΔCt^ method. The primer sequences are listed below:

5′-GGAGCGAGATCCCTCCAAAAT-3′ (forward) and

5′-GGCTGTTGTCATACTTCTCATGG-3′ (reverse) for GAPDH;

5′-CGAGGACAACGACTTCGTGTT-3′ (forward) and

5′-ACAATTTGCCGTAGGTAGTATCG-3′ (reverse) for PLK1.

### Western blotting (WB)

Total protein was extracted from the cultured cells and quantified. Equal amounts of protein (40 μg) per sample were separated by 10% SDS-PAGE and then transferred to PVDF membranes. After blocking, the membranes were incubated with a rabbit anti-PLK1 polyclonal antibody (A2548; Wuhan ABclonal Technology Co., Ltd., China), and then with a rabbit anti-goat secondary antibody (AS029). The protein bands were visualized using an enhanced chemiluminescence (ECL) reagent, and the signal intensities were quantified with GAPDH as the internal reference.

### Immunohistochemistry (IHC)

The *in-situ* expression of PLK1 in healthy breast tissues and tumor tissues was evaluated on the basis of the IHC data from the Human Protein Atlas (HPA, https://www.proteinatlas.org).

### Data processing and analysis

The “ImbTreeAUC” R package was used to plot the receiver operating characteristic (ROC) curves, and calculate area under curve (AUC) for predicting 1-, 3- and 5-year survival. The nomogram was constructed using the “rms” R package. Kaplan-Meier (K-M) survival curves were analyzed with the log-rank test. RF and SVM algorithms were used to construct the prognostic signature based on clinical data and PLK1 expression. Wilcoxon test was performed using the R software (version 4.2.1), and *p* ≤ 0.05 was considered statistically significant.

## Results

### PLK1 is overexpressed in BRCA and correlates with the clinicopathological features

**Figure 1:**
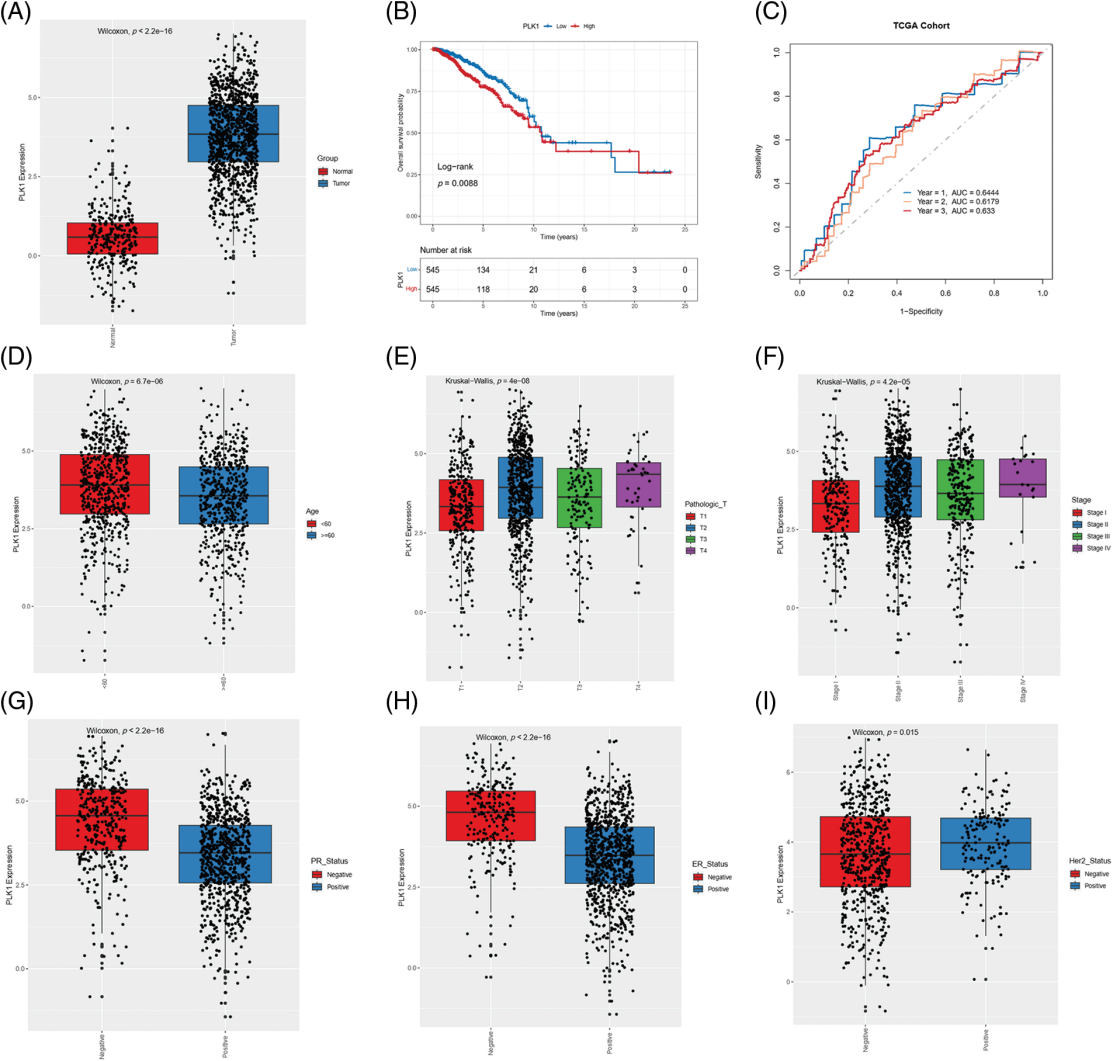
Correlation between PLK1 gene expression and clinical parameters. (A) Box plot showing the differential expression of PLK 1 in TCGA-BRCA samples. (B) Kaplan-Meier curves showing OS of patients demarcated by PLK1 expression level in the TCGA-BRCA cohort. (C) ROC curves of 1-, 3-, and 5-year OS on the basis of PLK1 expression. Box plots showing the correlation of PLK1 expression with (D) age, (E) T stage, (F) pathological stage, (G) PR status, (H) ER status and (I) Her-2 status.

PLK1 expression was significantly higher in the tumor tissues compared to the normal breast tissues in the TCGA-BRCA cohort (*p* < 2.2e−16; Fig. 1A), and correlated with lower survival rate (*p* = 0.0088; Fig. 1B). Furthermore, the AUC values of PLK1 for predicting 1-, 3- and 5-year overall survival (OS) of BRCA patients in this cohort were 0.6444, 0.6179, and 0.633, respectively (Fig. 1C). In addition, PLK1 expression was higher in the patients younger than 60 years of age (*p* = 6.7e−06), and those with advanced T stage (*p* = 4e−08) and pathological stage (*p* = 4.2e−05) tumors (Figs. 1D–1F). Furthermore, PLK1 was expressed at higher levels in the progesterone receptor (PR)-negative and estrogen receptor (ER)-negative tumors compared to the tumors positive for these receptors (Figs. 1G–1H). The median expression level of PLK1 in the Her-2-negative tumors was considerably lower than that in the Her-2-positive tumors, while the overall expression of negative status was higher than that of positive status (Fig. 1I). Taken together, these results suggested that high PLK1 expression in BRCA tissues portends worse prognosis.

### PLK1 is an independent risk factor for BRCA

**Figure 2:**
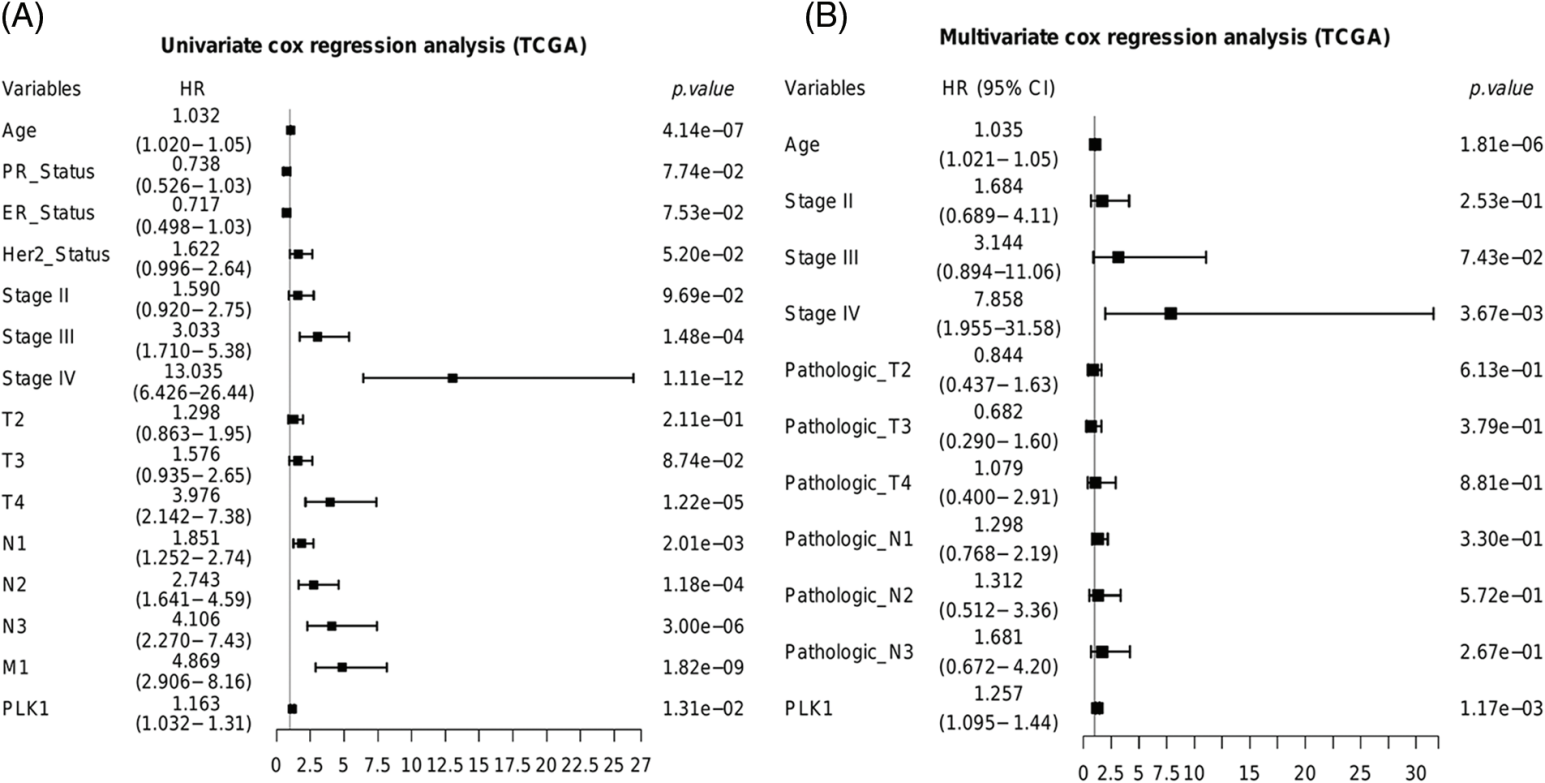
Predictive ability of PLK1 in the TCGA-BRCA cohort. Results of the (A) univariate and (B) multivariate COX forest plots of PLK1 and clinical features.

**Figure 3:**
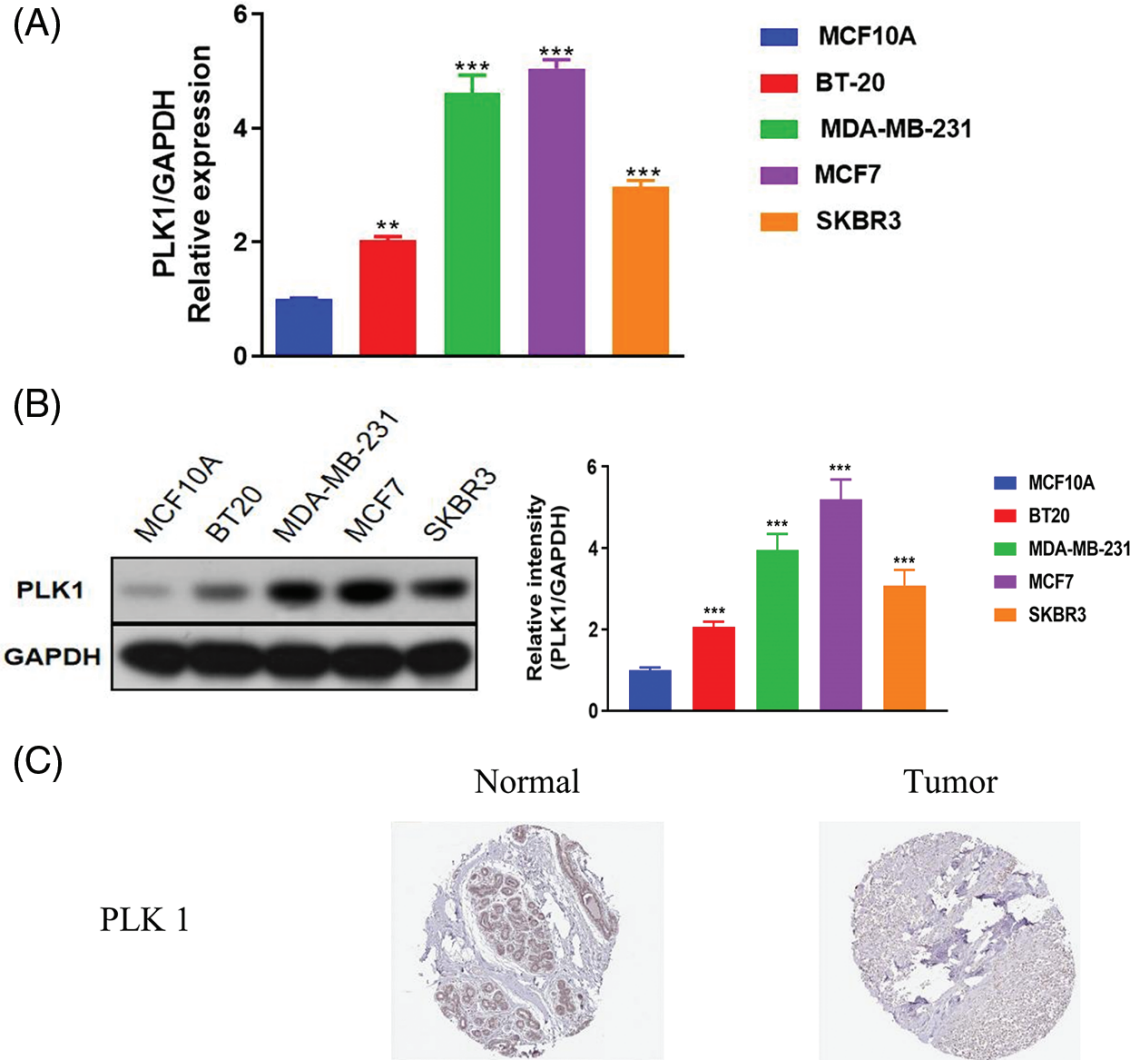
Validation of PLK1 mRNA and protein expression in BRCA. (A) PLK1 mRNA levels in the indicated cell lines. (B) PLK1 protein levels in the indicated cell lines. (C) IHC images from the Human Protein Atlas (HPA) database showing differential expression of PLK1 between the tumor and normal breast tissue samples. **p < 0.01, and ***p < 0.001.

The prognostic relevance of PLK1 was further evaluated by Cox regression analysis. As shown in Fig. 2A, PLK1 expression (*p* = 1.31e−02), age (*p* = 4.14e−07), pathological stage (stage III, *p* = 1.48e−04; stage IV, *p* = 1.11e−12), T stage (T4, *p* = 1.22e−05), N stage (N1, *p* =2.01e−03; N2, *p* = 1.18e−04; N3, *p* = 3.00e−06), and M stage (M1, *p* = 1.82e−09) were significantly correlated with the prognosis of BRCA according to univariate COX regression (Fig. 2A). Multivariate regression analysis further identified PLK1 expression (*p* = 1.17e−03), age (*p* = 1.81e−06) and pathological stage (stage IV, *p* = 3.67e−03) as independent risk factors for the OS of BRCA patients (Fig. 2B). Furthermore, PLK1 mRNA expression was lower in the normal breast epithelial cells (MCF10A) compared to that in the different breast cancer cell lines (BT-20, MCF-7, SK-BR-3 and MDA-MB-231) (Fig. 3A). The highest expression level of PLK1 was detected in the MCF-7 cells, indicating that PLK1 may play a greater role in invasive BRCA. Consistent with this, the PLK1 protein was also significantly upregulated in the breast cancer cell lines (Fig. 3B, *p* < 0.05). Likewise, analysis of the IHC images of healthy breast tissues and tumor tissues in HPA also showed a tendency of higher PLK1 expression in the tumors, although there was sample variability, as well as discrepancies in picture quality (Fig. 3C).

### PLK1 expression correlated to the immunotherapy response in BRCA

**Figure 4:**
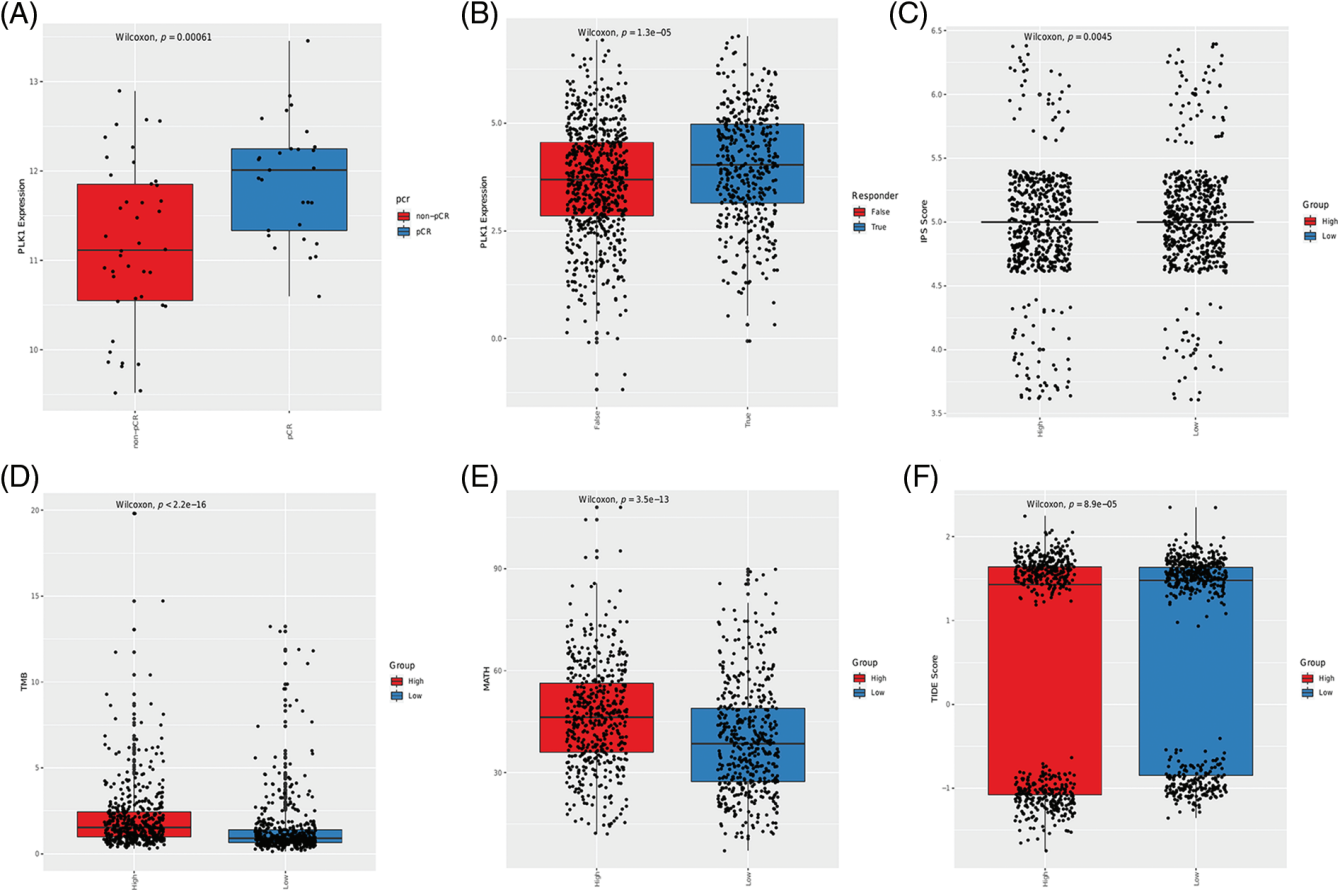
Association between immunotherapy response, immune-related scores and PLK1 expression in BRCA patients. (A) Box plot showing the correlation between PLK1 expression and pCR in the GSE173839 cohort. (B) Box plot showing the correlation between PLK1 expression and immunotherapy response. The IPS scores (C), TMB scores (D), MATH scores (E) and TIDE scores (F) in the PLK1-low and PLK1-high groups.

Although immune checkpoint inhibitors (ICIs) have shown encouraging results, only a small percentage of cancer patients benefit from immune checkpoint blockade therapies. Therefore, it is crucial to identify biomarkers in order to screen ideal candidates for immunotherapy. To this end, we assessed the correlation of PLK1 with TMB, MATH, IPS and TIDE scores to determine whether PLK1 can predict immunotherapy response. PLK1 expression was higher in the pCR group compared to the non-pCR group in the GSE173839 cohort (Fig. 4A). Furthermore, PLK1 was upregulated in the responders to immunotherapy (Fig. 4B). Based on the median expression of PLK1, the BRCA patients were divided into the PLK1-high and PLK1-low groups, which showed similar IPS scores (Fig. 4C). In contrast, the TMB and MATH scores were higher in the PLK1-high group (Figs. 4D and 4E), whereas the TIDE scores were higher for the PLK1-low patients (Fig. 4F).

### PLK1 is associated with the infiltration of immune cells in BRCA tumors

**Figure 5:**
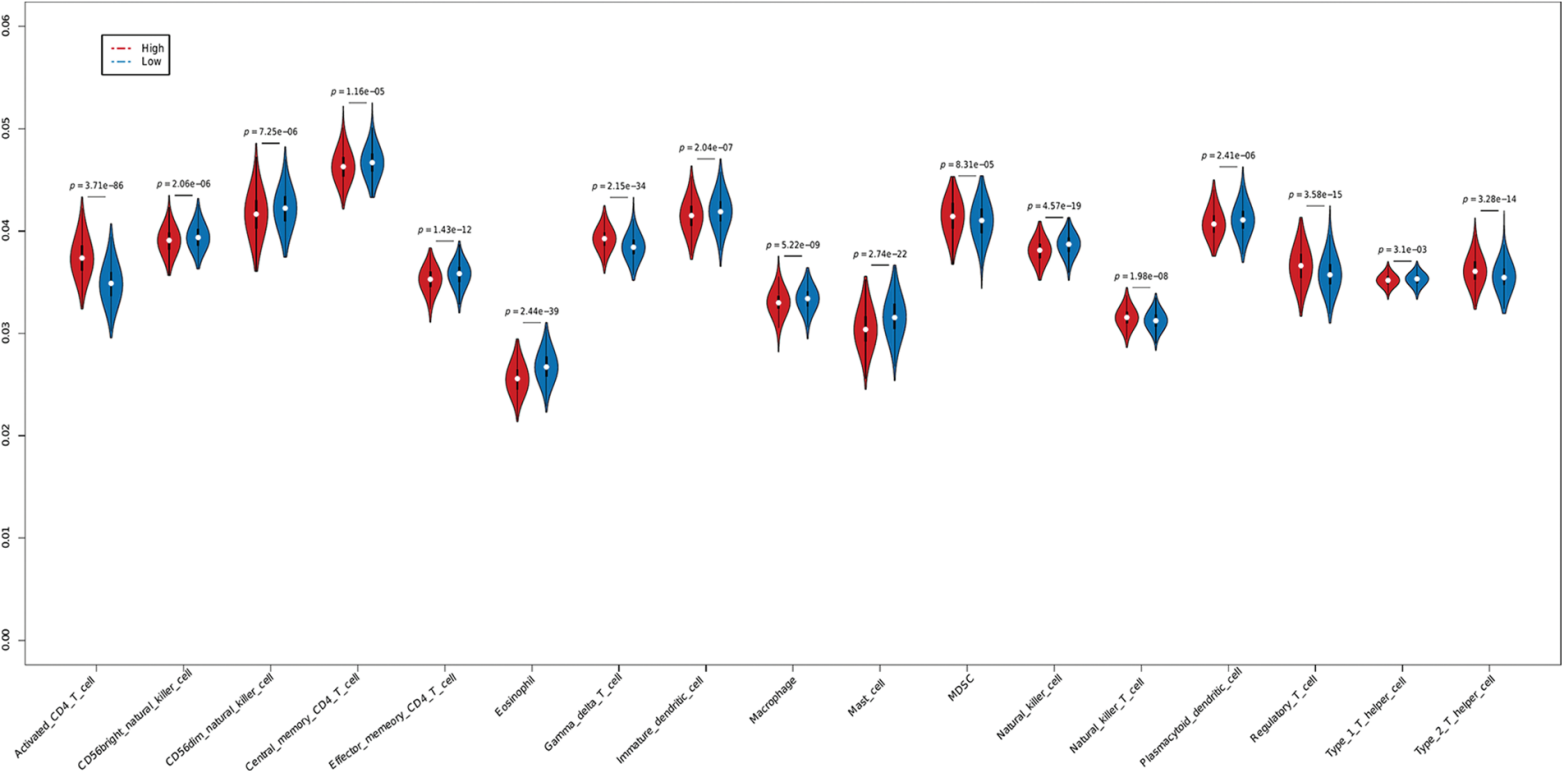
Immune infiltration in the PLK1-high and PLK1-low groups.

The tumor microenvironment (TME) is a key factor modulating cancer cell survival and metastasis [17]. In addition, the tumor-infiltrating immune cells determine the efficacy of immunotherapy. Given the correlation between PLK1 expression and immunotherapy response observed in the BRCA cohort, we next determined whether PLK1 is related to the infiltration of immune cells. The results of ssGSEA indicated similar proportions of the different immune cell populations in BRCA (Suppl. Figs. 2A and 2B). However, the infiltration of 17 immune cell types was associated with PLK1 expression. As shown in Fig. 5, the PLK1-high group had higher infiltration of 6 immune cell types and relatively low infiltration of 11 immune cell types. Notably, there was a higher abundance of tumor-promoting immune cells, such as Type 2 T helper cells, Myeloid-derived suppressor cells (MDSCs) and regulatory T cells, in the PLK1-high groups, which may be responsible for the worse prognosis.

### Identification of hub immune cells

**Figure 6:**
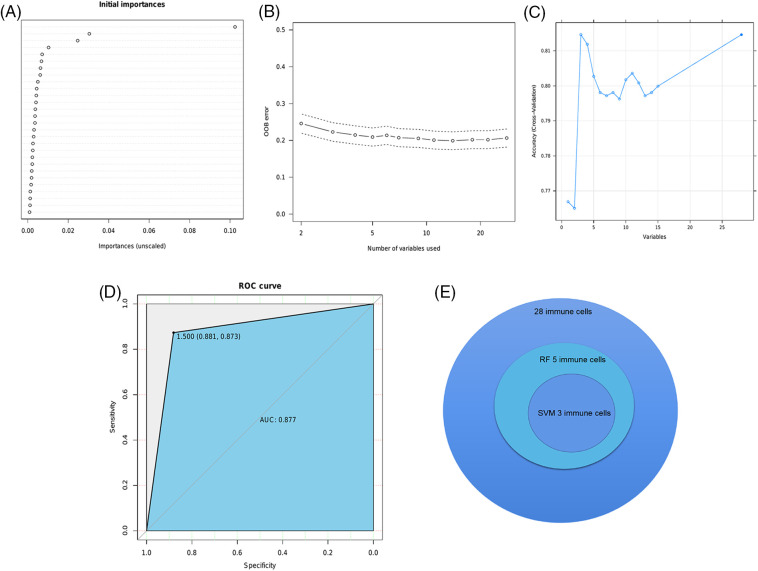
Identification of infiltrating immune cell populations. (A) The initial analysis of 28 immune cells by the RF algorithm. (B) The OOB error corresponding to the classification number. (C) The result of SVM algorithm. (D) The ROC curve of the model. (E) Three immune cell types were identified by RF and SVM algorithms.

To further identify the immune cells that are most relevant to the prognosis of BRCA, we performed SVM and RF analyses on 28 infiltrating immune cell populations. Five hub immune cells were identified by the RF analysis (Figs. 6A and 6B), of which gamma delta (γδ) T cells, eosinophils and activated CD4 T cells were also identified in the SVM method (Fig. 6C, Table 1). The AUC of this model was 0.877 (Fig. 6D). The overlapping immune cell types are shown in Fig. 6E.

**Table 1:** RF analysis and SVM analysis of candidate immune cells

Immune cells	MeanDecreaseAccuracy
Activated CD4 T cell ^✳^	0.1025
Gamma delta T cell ^✳^	0.0296
Eosinophil ^✳^	0.025
Natural killer cell	0.0103
Mast cell	0.0074

Note: ✳ Immune cells were also the result of the SVM analysis.

### Risk score based on immune infiltration and PLK1 expression predicted survival of BRCA patients

**Figure 7:**
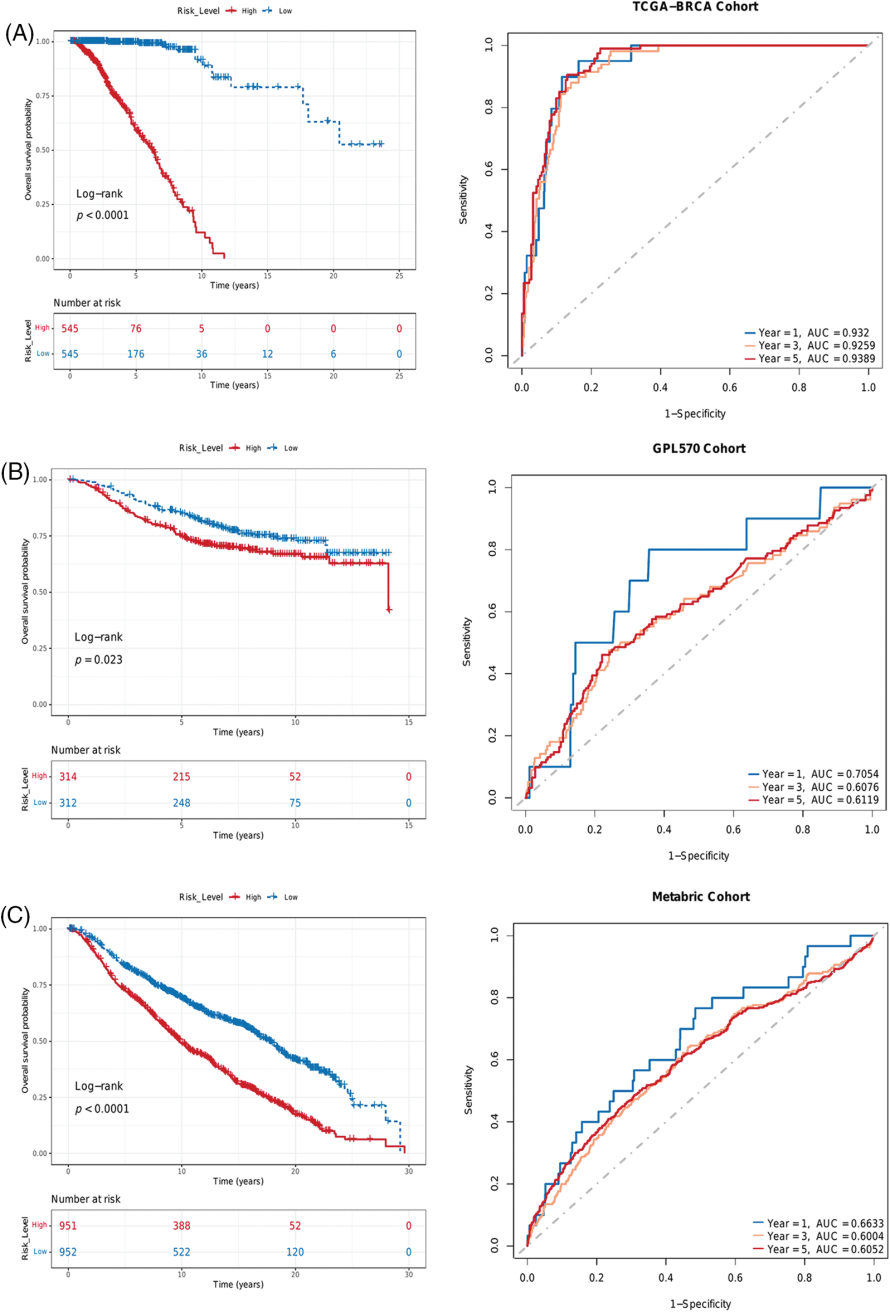
Risk score based on PLK 1 expression and immune infiltration predicted survival in different BRCA datasets. Kaplan-Meier survival curves of the two risk groups and ROC curves of the risk score in the (A) TCGA, (B) GEO and (C) METABRIC datasets.

Given the low sensitivity of immunotherapy and the heterogeneity of TME, we devised a risk score based on PLK1 expression and the infiltration of γδ T cells, eosinophils and activated CD4 T cells to predict the prognosis of BRCA patients. The risk score was calculated using the RF algorithm, and the patients were stratified into the high-risk and low-risk groups according to the median risk score. Lower risk scores were associated with higher survival rates in the TCGA-BRCA, GEO and METABRIC datasets (*p* < 0.0001, Fig. 7A; *p* = 0.023, Fig. 7B; *p* < 0.0001, Fig. 7C). Furthermore, the AUC values of the risk score in these datasets were also higher than 0.6 (Figs. 7A–7C), indicating that it can predict the prognosis of BRCA patients.

### Identification of independent prognostic factors for BRCA

**Figure 8:**
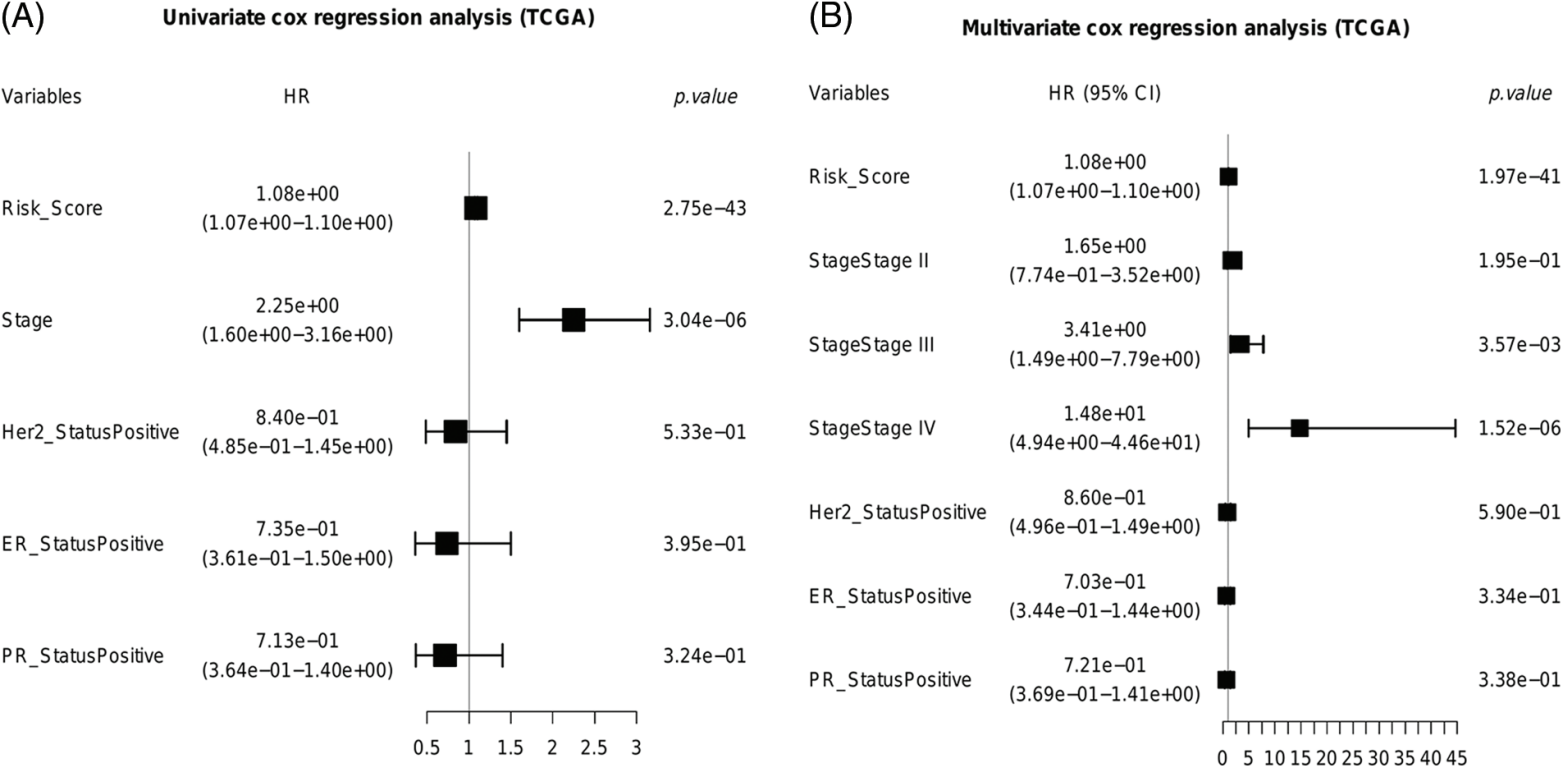
Identification of independent prognostic factors for BRCA. (A) The univariate cox forest plot. (B) The multivariable cox forest plot.

We next identified the prognostic factors of BRCA by Cox regression analysis. As shown in Fig. 8A, the pathological stage (HR = 2.25e+00, 95% CI:1.60e+00–3.16e+00, *p* = 3.04e−06) and risk score (HR = 1.08e+00, 95% CI:1.07e+00–1.10e+00, *p* = 2.75e−43) were significantly associated with the prognosis. Furthermore, the risk score (HR = 1.08e+00, 95% CI:1.07e+00–1.10e+00, *p* = 1.97e−41) and advanced stage (Stage III, HR = 3.41e+00, 95% CI:1.49e+00–7.79e+00, *p* = 3.57e−03; Stage IV, HR = 1.48e+01, 95% CI:4.94e+00–4.46e+01, *p* = 1.52e−06) were identified as independent prognostic factors for BRCA (Fig. 8B).

### Construction and validation of a predictive nomogram for BRCA

**Figure 9:**
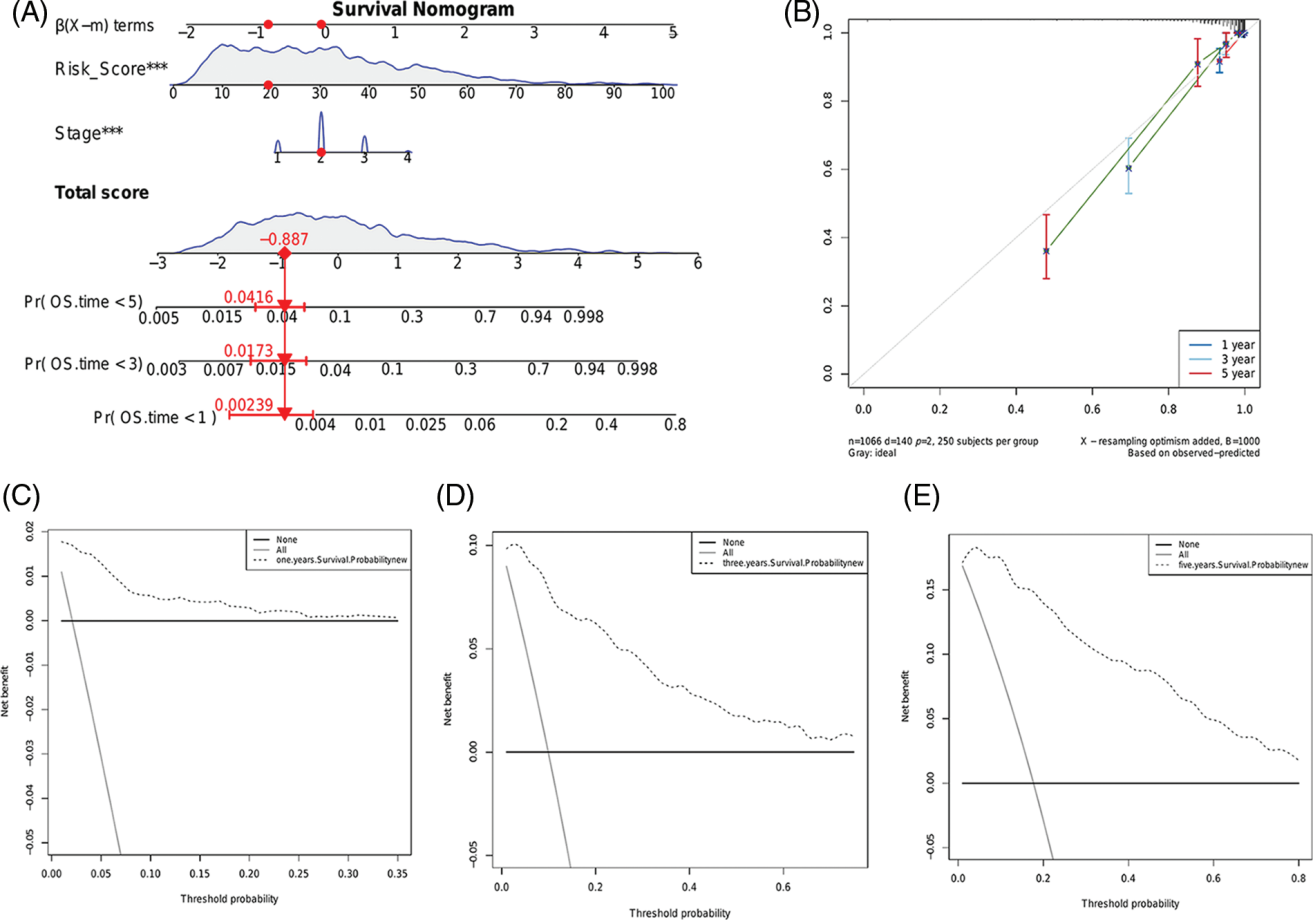
Construction and evaluation of a predictive nomogram. (A) Nomogram consisting of the risk score and pathological stage to predict the survival of BRCA patients. (B) Probability of 1-, 3- or 5-year survival as predicted by the nomogram and calibration curve showing the consistency. The DCA of the nomogram for predicting 1- (C), 3- (D) and 5-year (E) overall survival.

We constructed a nomogram using the risk score and pathological stage to predict the survival of BRCA patients in a clinical setting (Fig. 9A). The scores of each prognostic factor were summed to obtain the total score, and a higher total score was indicative of worse prognosis. The calibration curve showed good predictive ability of the nomogram in the entire cohort (Fig. 9B). Furthermore, decision curve analysis (DCA) showed that the nomogram can benefit BRCA patients in clinical practice (Figs. 9C–9E).

### PLK1 expression can predict the response to immunotherapy

**Figure 10:**
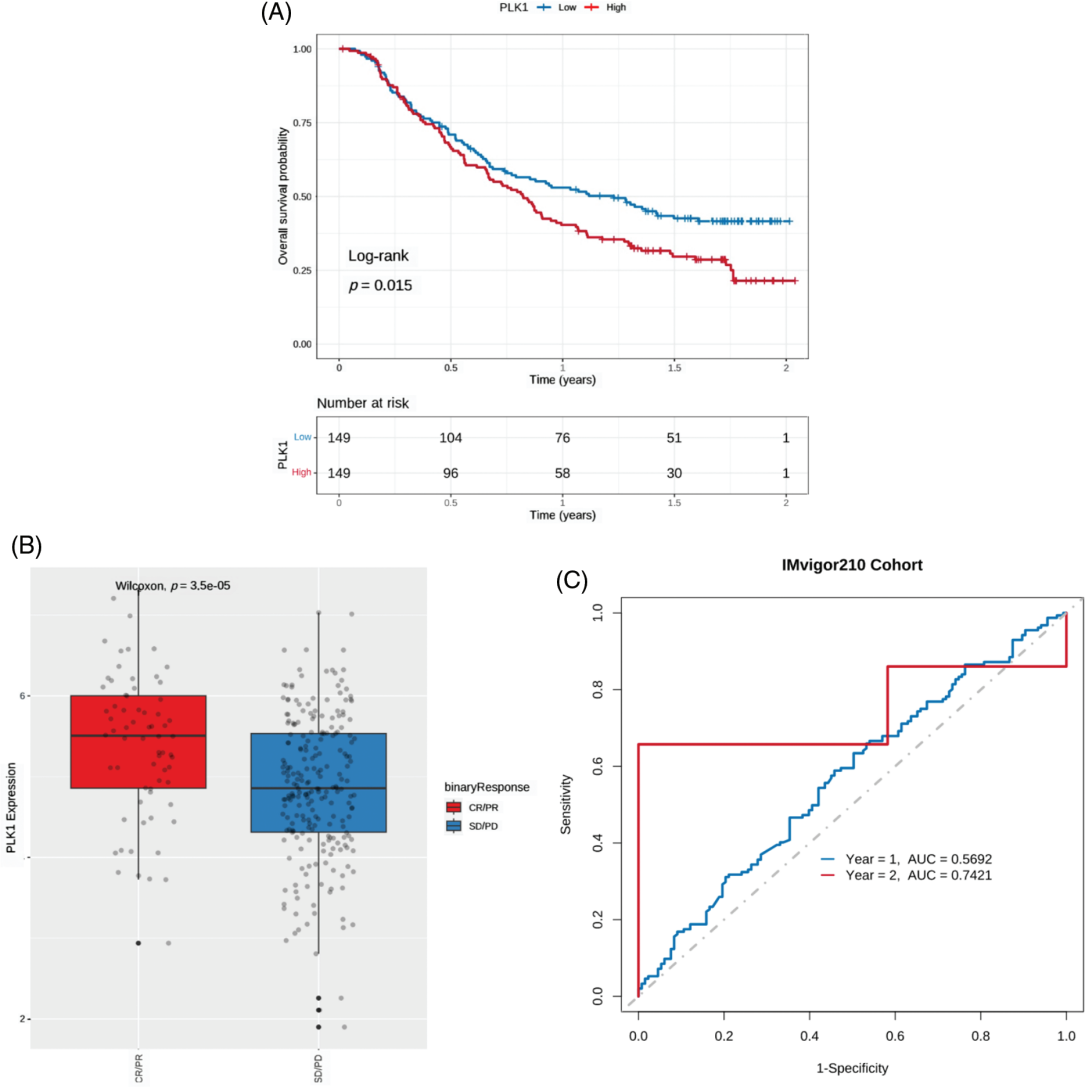
PLK1 expression can predict the benefits of immunotherapy. (A) Kaplan-Meier survival curves of PLK1-high and PLK1-low urothelial cancer patients in the lMvigor210 cohort. (B) Boxplot showing PLK1 expression in patients with different immunotherapy responses in the IMvigor210 cohort. CR: complete response, PR: partial response, and SD: stable disease, PD: partial response. (C) ROC curves showing the predictive ability of PLK1 for 1- and 2-year survival post-immunotherapy in the IMvigor210 cohort.

Due to the lack of publicly available survival data of BRCA patients who received immunotherapy, we used the IMvigor210 dataset of urothelial cancer patients with anti-PD-L1 therapy to determine whether PLK1 expression can predict immunotherapeutic benefit. As shown in the survival curves in Fig. 10A, patients with the low PLK1 expression had significantly better prognosis compared to those with high PLK1 expression. Furthermore, PLK1 expression was significantly higher in patients with complete or partial response compared to those with stable or progressive disease (Fig. 10B). The AUC values of the prognostic model for 1-year and 2-year OS in the IMvigor210 cohort were 0.5692 and 0.7421, respectively (Fig. 10C).

## Discussion

Breast cancer is the second most commonly diagnosed cancer in women [18], and the incidence rate of BRCA has increased over the last 15 years [19]. While overall prognosis continues to be unsatisfactory, early detection and advances in treatment strategies have significantly reduced BRCA-related deaths [20]. Therefore, it is crucial to explore novel biomarkers for the prognostic assessment of BRCA.

The PLK family of proteins control mitotic termination by regulating the production of anaphase-promoting complexes and coordinating cytokinesis [21]. PLK3 has been previously identified as a novel independent prognostic marker for breast cancer, indicating a role of this isoform in disease progression. Therefore, PLK proteins are promising targets for the development of new anti-cancer drugs [22]. PLK1 is normally enriched in the mitotic centrosome, centromeres, and cytokinesis intermediates, and regulates mitosis by phosphorylating specific downstream targets [23]. Previous studies have shown that anti-sense inhibitors targeting PLK1 can sensitize BRCA cells to chemotherapy drugs [24]. Furthermore, there is evidence that PLK1 regulates the transcription of ER in human breast cancer cells [25], and is associated with the prognosis and survival of BRCA patients [26].

In this study, we observed a significant upregulation of PLK1 in the BRCA tissues compared to the healthy breast tissues in three datasets. Furthermore, high PLK1 expression correlated with worse prognosis in the BRCA patients, and showed good predictive performance for patient survival. In addition, PLK1 expression level, age and the tumor stage were identified as independent prognostic markers for BRCA. Consistent with these results, PLK1 expression level was higher in multiple breast cancer cell lines compared to normal breast epithelial cells, especially in the invasive breast cancer cell line (MCF-7). Likewise, analysis of IHC images in the HPA database revealed that PLK1 expression was higher in the breast tumor tissues compared to the normal tissues. These findings suggested that PLK1 may play a significant role in invasive breast cancer.

To determine whether PLK1 can predict immunotherapy response in BRCA, we evaluated its correlation with different clinical indicators. In the GSE173839 cohort, PLK1 expression was higher in the pCR group compared to the non-pCR group, as well as in the immunotherapy responders. Furthermore, high PLK1 expression was associated with higher TMB and MATH scores, and lower TIDE scores. A higher TMB score correlates with better immunotherapeutic outcomes, whereas higher MATH scores are indicative of more pronounced tumor heterogeneity. However, a higher TIDE score corresponds to increased possibility of immune escape, and lower success rate of immunotherapy. Accordingly, we surmised that high PLK1 expression in BRCA tissues can improve the efficacy of immunotherapy on account of increased tumor heterogeneity. However, the higher expression of PLK1 observed in the pCR group contradicts the above hypothesis. One explanation is that pCR may only reflect the status of primary and/or axillary lymph node metastases after neoadjuvant treatment, and not of distant metastases.

The ssGSEA algorithm was used to evaluate the immune infiltration. High PLK1 expression was associated with increased infiltration of 6 immune cell types and lower infiltration state of 11 immune cell types. Furthermore, RF and SVM algorithms identified γδ T cells, eosinophils and activated CD4 T cells as the major infiltrating immune cells in BRCA. The CD8 T cells are the predominant tumor-infiltrating leukocytes in breast cancer, followed by macrophages, regulatory T cells, CD4 T cells, etc. [27], and the T cell subsets are involved in immune surveillance [28]. The primed CD8 and CD4 T cells can directly target and kill cancer cells [29], and one of the components of the cancer immune cycle is the interference between these T lymphocytes [30]. Studies show that increased infiltration of CD4 T cells is often predictive of a better survival outcome and favorable prognosis of breast cancer patients [31]. Likewise, CD4 T cell infiltration is a favorable prognostic factor in various tumors [32–34], and high intra-tumoral density of CD4 T cells is associated with good clinical outcomes [35,36]. In one meta-analysis, infiltration of eosinophils was correlated to a favorable prognosis in multiple cancer types [37]. Another study examined eosinophil infiltration in distinct tumor types, and found lowest infiltration in BRCA and highest in gastrointestinal cancer [38]. Furthermore, there is evidence that eosinophils are involved in the immune response to breast tumors, and the eosinophil gene signature in tumor biopsies is associated with the response to immunotherapy, although the exact molecular mechanisms are unknown [39]. The γδ T cells have both immune effector and regulatory functions [40,41], and are known to exert both anti-cancer and pro-tumorigenic effects in the TME [42]. One study showed that depletion of γδ T cells in the TME creates an immunosuppressive milieu that promotes drug resistance [43]. Nevertheless, infiltration of γδ T cells in pancreatic, colon and breast tumors has been related to poor prognosis, indicating that this subset can also promote tumor growth [44]. Overall, our findings suggest that γδ T cells and activated CD4 T cells correlate with favorable prognosis in BRCA, whereas high infiltration of eosinophils portends poor prognosis.

We devised a prognostic risk score for BRCA based on PLK1 expression and the infiltration of hub immune cells, and found that higher risk scores were associated with worse survival in three different BRCA datasets. Furthermore, the pathological stage and risk score were identified as independent predictors of survival, which is consistent with the correlation between PLK1 expression and pathological stage reported previously [19]. In addition, PLK1 is a potential drug target on account of its high expression in advanced breast cancer stages. A nomogram was constructed by combining the risk score and pathological stage, which showed good predictive performance and potential clinical benefit for BRCA patients. Although the clinical outcomes of anti-PD-1 or anti-PD-L1 antibodies against solid tumors have been encouraging, only a small percentage of cancer patients have benefited from immunotherapy. Therefore, it is crucial to identify biomarkers to screen for patients who are most likely to benefit from immunotherapy. PLK1 expression effectively predicted the outcomes in urothelial carcinoma patients in the IMvigor210 cohort after anti-PD-L1 therapy, and thus warrants further exploration as a predictive biomarker of immunotherapy response.

## Conclusion

PLK1 is a promising prognostic biomarker for BRCA, and the risk score combining PLK1 expression and immune infiltration can predict the response to immunotherapy. Furthermore, the nomogram comprising of the risk score and pathological stage showed good predictive performance for the survival of BRCA patients, and should be further validated in clinical trials.

## Supplementary Materials

Supplementary Figure S1 The flowchart of this study.

Supplementary Figure S2 The infiltrated situation in the BRCA microenvironment was evaluated on the basis of ssGSEA algorithm. (A) The percentage of cells infiltration. (B) Heatmap of cells infiltration.

## Data Availability

The Cancer Genome Atlas (TCGA, https://portal.gdc.cancer.gov/); The Gene Expression Omnibus (GEO, https://www.ncbi.nlm.nih.gov/geo); The cBio cancer genomics portal (cBioPortal, http://www.cbioportal.org); “randomForestSRC” R package (https://www.randomforestsrc.org/index.html).

## Supplementary Materials

The supplementary material is available online at https://doi.org/10.32604/or.2023.030887.
