# Supplementary figures and images for: Polo-like kinase 1 as a biomarker predicts the prognosis and immunotherapy of breast invasive carcinoma patients
Source: Oncol Res. 2023 Dec 28;32(2):339–51. doi: 10.32604/or.2023.030887 (PMC10765123; doi:10.32604/or.2023.030887)

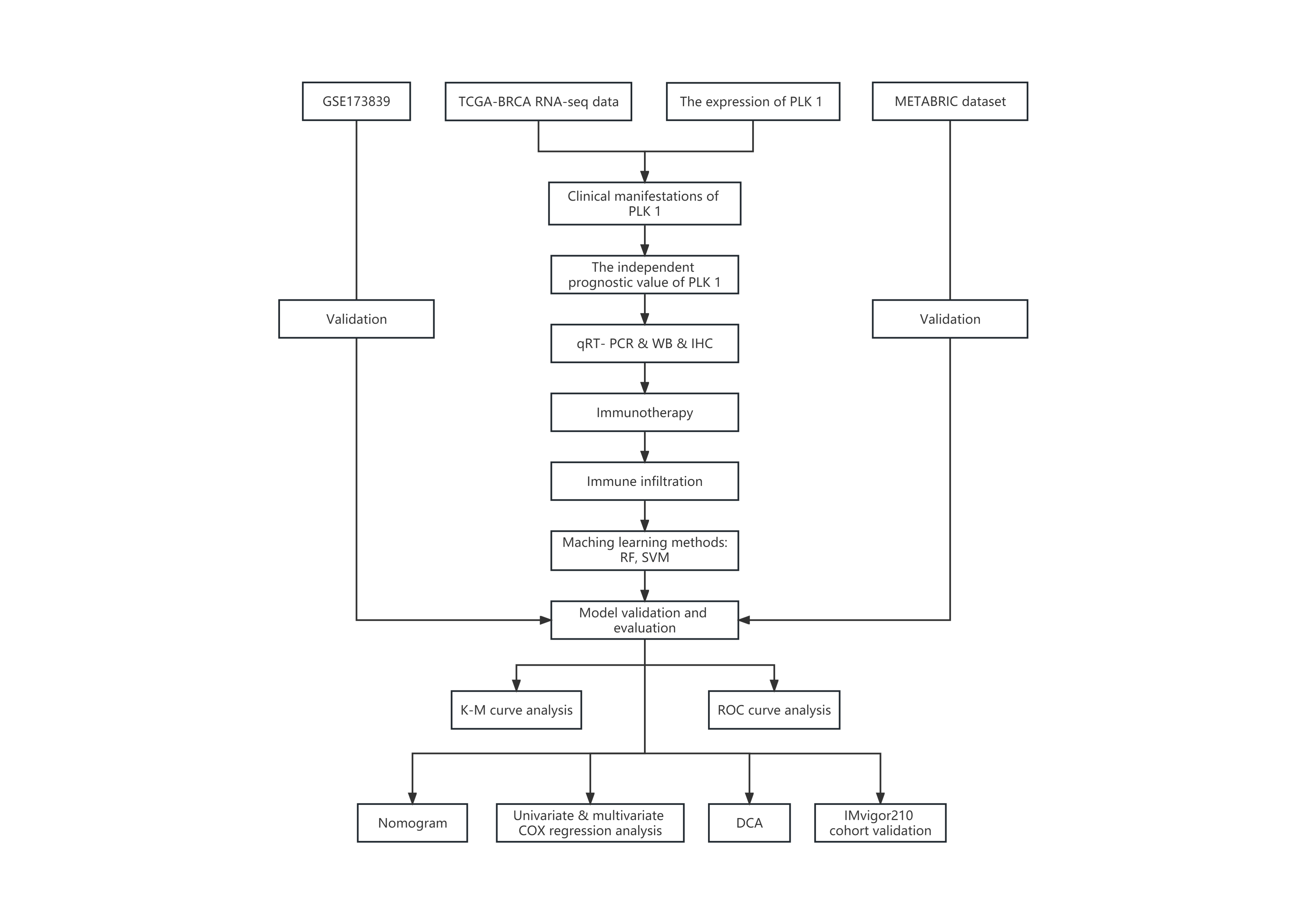

Supplement: Supplementary Figure S1 [file OncolRes-32-30887-s001.tif]

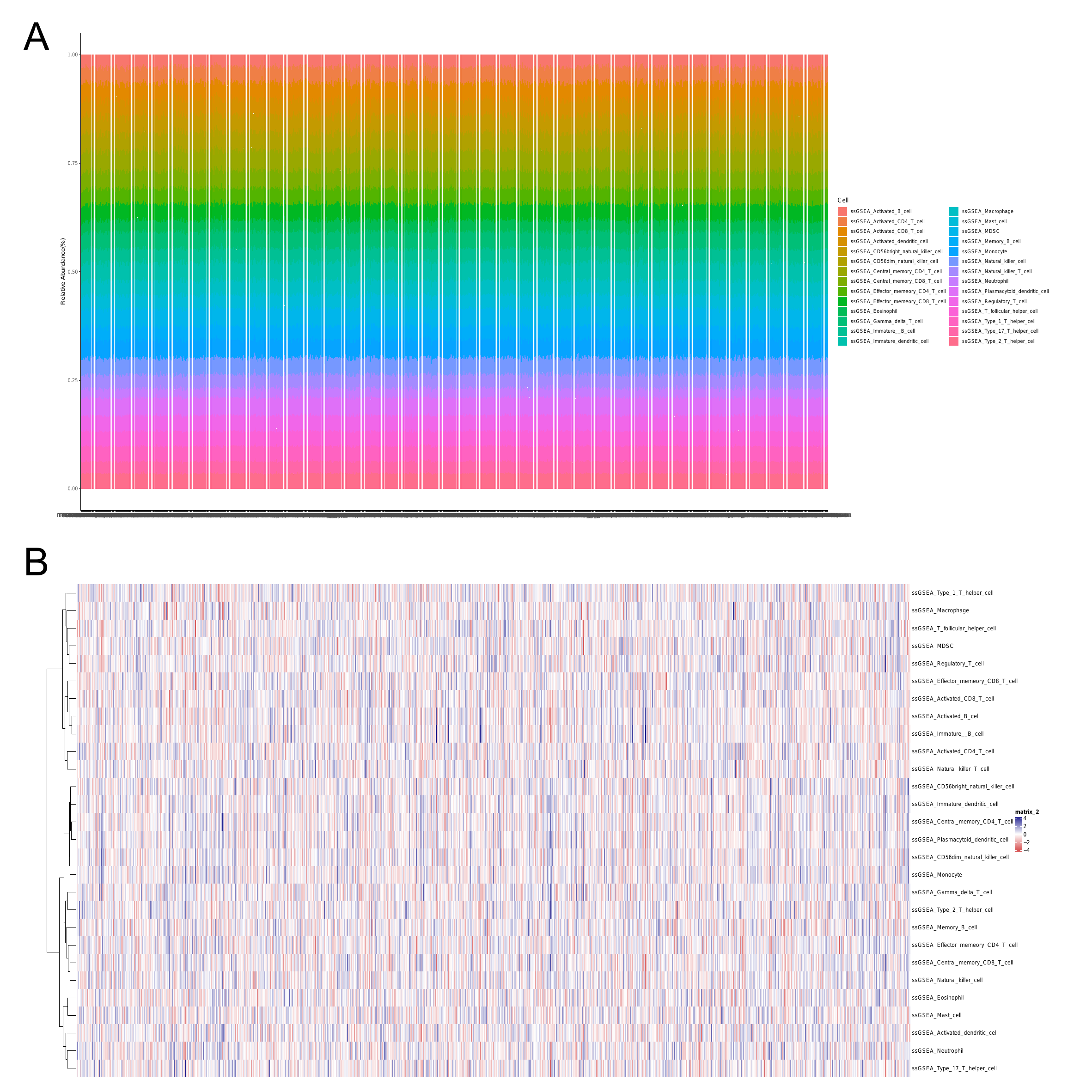

Supplement: Supplementary Figure S2 [file OncolRes-32-30887-s002.tif]
